# Supplementary material for: Influence of cognitive demand and auditory noise on postural dynamics
Source: Exp Brain Res. 2025 Feb 1;243(2):54. doi: 10.1007/s00221-025-06998-w (PMC11787163; doi:10.1007/s00221-025-06998-w)
Supplement: Supplementary file 1 — Supplementary file1 (DOCX 335 KB) [file 221_2025_6998_MOESM1_ESM.docx]

Influence of Cognitive Demand and auditory noise on Postural Dynamics

*Sam Carey^1^ & Ramesh Balasubramaniam^1^

1 Cognitive & Information Sciences, University of California, Merced, CA, United States

Key Words: Postural Sway; Auditory Feedback; White Noise; Cognitive Load; N-back; Working Memory

Address for Correspondence:

Sam Carey

Sensorimotor Neuroscience Laboratory

Cognitive & Information Sciences

University of California, Merced

5200 N Lake Road, Merced, CA 95343 USA

<http://www.rameshlab.com>

Email: scarey5@ucmerced.edu

Supplementary Information

Table 1: Radial Sway Post Hoc Comparisons

| Contrasts | Estimate | SE | T-Ratio | P Value |
| --- | --- | --- | --- | --- |
| None Silent – Easy Silent | -0.1444 | -0.199 | -0.726 | 0.9969 |
| None Silent – Hard Silent | -0.7532 | -0.199 | -3.786 | 0.0023 |
| None Silent – None Noise | 1.2728 | 0.203 | 6.281 | <.0001 |
| None Silent – Easy Noise | 0.2979 | 0.200 | 1.493 | 0.6690 |
| None Silent – Hard Noise | 0.0613 | 0.200 | 0.307 | 0.9996 |
| Easy Silent – Hard Silent | -0.6087 | 0.195 | -3.144 | 0.0234 |
| Easy Silent – None Noise | 1.4172 | 0.200 | 7.095 | <.0001 |
| Easy Silent – Easy Noise | 0.4423 | 0.196 | 2.255 | 0.2142 |
| Easy Silent – Hard Noise | 0.2057 | 0.196 | 1.048 | 0.9013 |
| Hard Silent – None Noise | 2.0259 | 0.200 | 10.133 | <.0001 |
| Hard Silent – Easy Noise | 1.0510 | 0.196 | 5.356 | <.0001 |
| Hard Silent – Hard Noise | 0.8145 | 0.196 | 4.151 | 0.0005 |
| None Noise – Easy Noise | -0.9749 | 0.201 | -4.862 | <0.0001 |
| None Noise – Hard Noise | -1.2115 | 0.201 | -6.035 | <0.0001 |
| Easy Noise – Hard Noise | -0.2366 | 0.197 | -1.201 | 0.8363 |

Table 2: High-Frequency RS Post Hoc Comparison

| Contrasts | Estimate | SE | T-Ratio | P Value |
| --- | --- | --- | --- | --- |
| None Silent – Easy Silent | 0.0182 | 0.0755 | 0.242 | 0.9999 |
| None Silent – Hard Silent | -.3232 | 0.0760 | -4.251 | 0.0004 |
| None Silent – None Noise | 0.3472 | 0.0767 | 4.527 | 0.0001 |
| None Silent – Easy Noise | 0.1664 | 0.0758 | 2.195 | 0.2415 |
| None Silent – Hard Noise | -0.1335 | 0.0758 | -1.762 | 0.4909 |
| Easy Silent – Hard Silent | -0.3414 | 0.0746 | -4.578 | 0.0001 |
| Easy Silent – None Noise | 0.3290 | 0.0755 | 4.357 | 0.0002 |
| Easy Silent – Easy Noise | 0.1482 | 0.0743 | 1.994 | 0.3467 |
| Easy Silent – Hard Noise | -0.1518 | 0.0743 | -2.041 | 0.3200 |
| Hard Silent – None Noise | 0.6704 | 0.0760 | 8.816 | <.0001 |
| Hard Silent – Easy Noise | 0.4896 | 0.0749 | 6,537 | <.0001 |
| Hard Silent – Hard Noise | 0.1897 | 0.0747 | 2.540 | 0.1142 |
| None Noise – Easy Noise | -.1808 | 0.0758 | -2.383 | 0.1636 |
| None Noise – Hard Noise | -0.4807 | 0.0758 | -6.344 | <.0001 |
| Easy Noise – Hard Noise | -0.3000 | 0.0747 | -4.016 | 0.0009 |

Table 3: Low-Frequency RS Post Hoc Comparisons:

| Contrasts | Estimate | SE | T-Ratio | P Value |
| --- | --- | --- | --- | --- |
| None Silent – Easy Silent | 0.0867 | 0.181 | 0.479 | 0.9969 |
| None Silent – Hard Silent | -0.5236 | 0.180 | -2.903 | 0.0441 |
| None Silent – None Noise | 1.0728 | 0.184 | 5.826 | <.0001 |
| None Silent – Easy Noise | 0.5048 | 0.182 | 2.774 | 0.0629 |
| None Silent – Hard Noise | 0.3494 | 0.181 | 1.933 | 0.3827 |
| Easy Silent – Hard Silent | -0.6103 | 0.178 | -3.437 | 0.0082 |
| Easy Silent – None Noise | 0.9860 | 0.182 | 5.418 | <.0001 |
| Easy Silent – Easy Noise | 0.4180 | 0.179 | 2.335 | 0.1816 |
| Easy Silent – Hard Noise | 0.2627 | 0.178 | 1.477 | 0.6791 |
| Hard Silent – None Noise | 1.5963 | 0.181 | 8.814 | <.0001 |
| Hard Silent – Easy Noise | 1.0283 | 0.178 | 5.764 | <.0001 |
| Hard Silent – Hard Noise | 0.8730 | 0.177 | 4.928 | <.0001 |
| None Noise – Easy Noise | -0.5680 | 0.183 | -3.107 | 0.0241 |
| None Noise – Hard Noise | -0.7234 | 0.182 | -3.983 | 0.0011 |
| Easy Noise – Hard Noise | -0.1553 | 0.179 | -0.869 | 0.9536 |

Table 4: DFA Post Hoc Comparisons:

| Contrasts | Estimate | SE | T-Ratio | P Value |
| --- | --- | --- | --- | --- |
| None Silent – Easy Silent | 0.01948 | 0.0115 | 1.695 | 0.5356 |
| None Silent – Hard Silent | 0.03108 | 0.0116 | 2.686 | 0.0791 |
| None Silent – None Noise | 0.09826 | 0.0117 | 8.363 | <.0001 |
| None Silent – Easy Noise | 0.05501 | 0.0116 | 4.738 | <.0001 |
| None Silent – Hard Noise | 0.05669 | 0.0116 | 4.899 | <.0001 |
| Easy Silent – Hard Silent | 0.01160 | 0.0114 | 1.016 | 0.9126 |
| Easy Silent – None Noise | 0.07878 | 0.0116 | 6.766 | <.0001 |
| Easy Silent – Easy Noise | 0.03553 | 0.0115 | 3.102 | 0.0243 |
| Easy Silent – Hard Noise | 0.03721 | 0.0114 | 3.259 | 0.0148 |
| Hard Silent – None Noise | 0.06718 | 0.0117 | 5.735 | <.0001 |
| Hard Silent – Easy Noise | 0.02393 | 0.0115 | 2.076 | 0.3012 |
| Hard Silent – Hard Noise | 0.02561 | 0.0115 | 2.232 | 0.2245 |
| None Noise – Easy Noise | -0.04325 | 0.0118 | -3.681 | 0.0034 |
| None Noise – Hard Noise | -0.04157 | 0.0117 | -3.546 | 0.0055 |
| Easy Noise – Hard Noise | 0.00168 | 0.0115 | 0.146 | 1.0000 |

Supplementary Plots: Analysis plots showing comparisons between silent and noise stimulation on the left with all cognitive conditions averaged across for their respective stimulation input. Radial sway plots of the cognitive load conditions, with the noise stimulation averaged across their respective cognitive task conditions.

Radial Sway


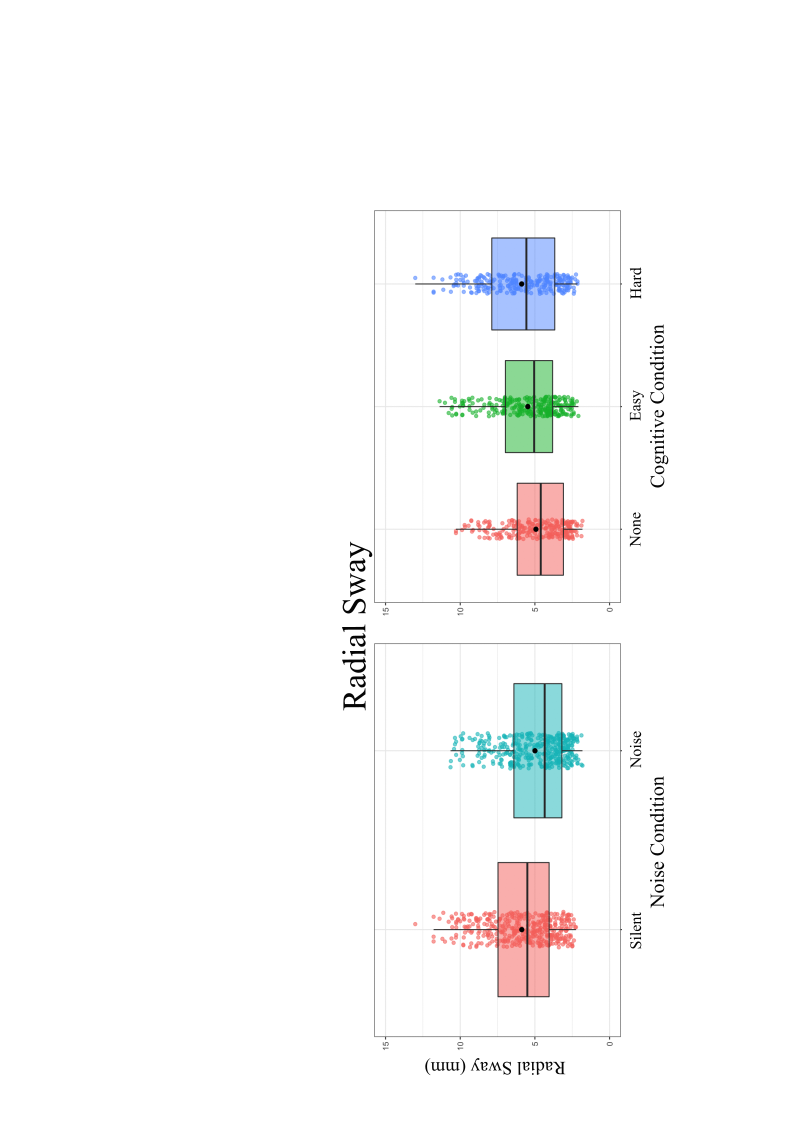


Supplementary Figure 1. Plot on the left show all noise conditions and silence conditions averaged across the cognitive task conditions to better visualize the effect of noise on Radial Sway while the plot on the right did the opposite, averaging across the noise conditions to visualize the effect of the cognitive load on Radial Sway. Box and whiskers plot with the solid black line representing the median, the solid black dot representing the mean, and the extending lines showing the maximum and minimum values.

High-Frequency Radial Sway


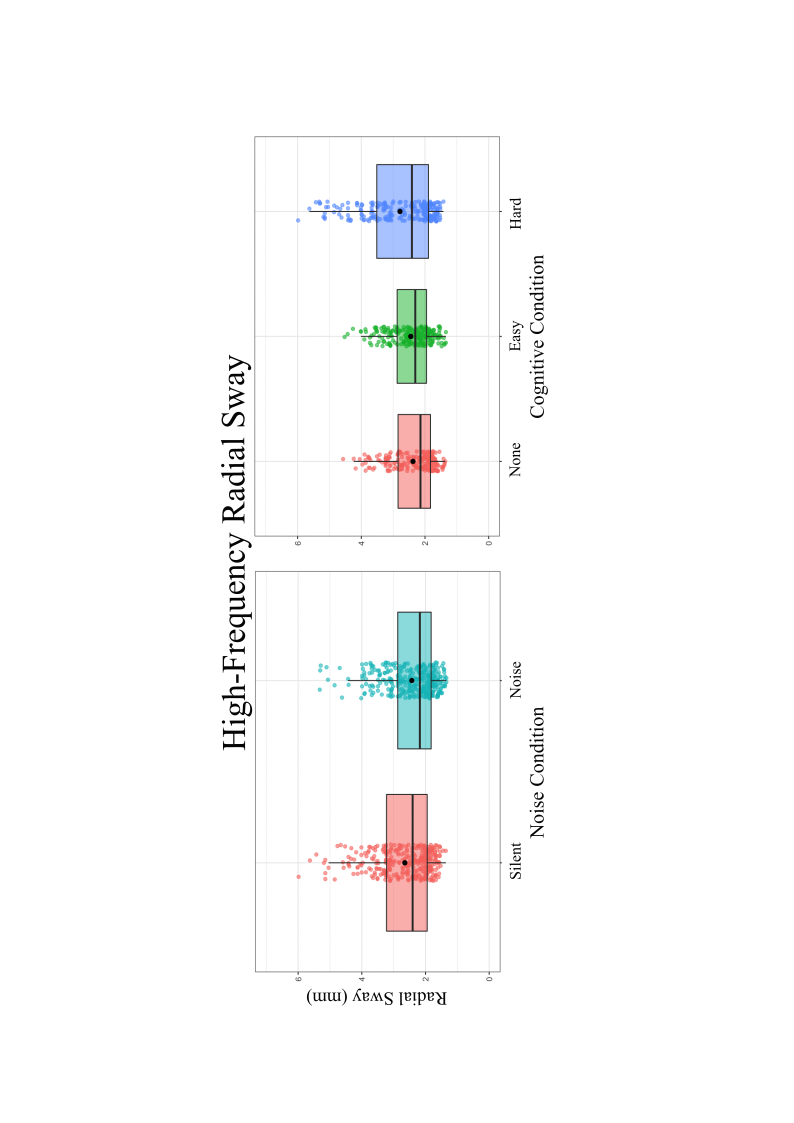


Supplementary Figure 2. Plot on the left show all noise conditions and silence conditions averaged across the cognitive task conditions to better visualize the effect of noise on High-Frequency Radial Sway while the plot on the right did the opposite, averaging across the noise conditions to visualize the effect of the cognitive load on High-Frequency Radial Sway. Box and whiskers plot with the solid black line representing the median, the solid black dot representing the mean, and the extending lines showing the maximum and minimum values.

Low-Frequency Radial Sway


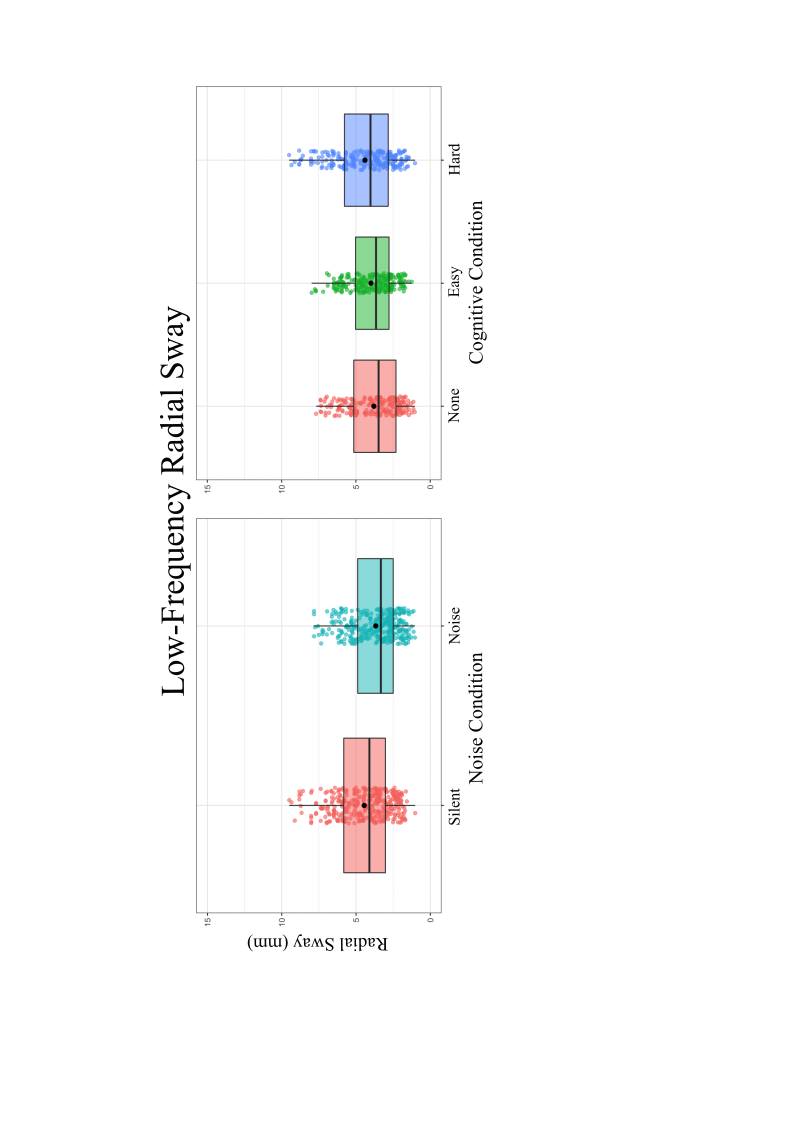


Supplementary Figure 3. Plot on the left show all noise conditions and silence conditions averaged across the cognitive task conditions to better visualize the effect of noise on Low-Frequency Radial Sway while the plot on the right did the opposite, averaging across the noise conditions to visualize the effect of the cognitive load on Low-Frequency Radial Sway. Box and whiskers plot with the solid black line representing the median, the solid black dot representing the mean, and the extending lines showing the maximum and minimum values.
